# Public Awareness and Beliefs About Complementary and Alternative Medicine and Holistic Health in Jordan: A Cross-Sectional Survey

**DOI:** 10.3390/healthcare14213629

**Published:** 2026-10-26

**Authors:** Kanar Sweiss, Abdallah Y. Naser, Alaa A. Alsharif

**Affiliations:** 1 Department of Basic Pharmaceutical Sciences, Faculty of Pharmacy, Middle East University (MEU), Amman 11831, Jordan; 2 Department of Applied Pharmaceutical Sciences and Clinical Pharmacy, Faculty of Pharmacy, Isra University, Amman 11622, Jordan; 3 Department of Pharmacy Practice, College of Pharmacy, Princess Nourah bint Abdulrahman University, P.O. Box 84428, Riyadh 11671, Saudi Arabia

**Keywords:** awareness, beliefs, complementary, alternative medicine, Holistic health, general public, Jordan

## Abstract

Background: Complementary and alternative medicine (CAM) is widely used, often without medical supervision, yet there are limited studies on public beliefs about CAM in Jordan. This study aimed to assess awareness of CAM and beliefs about CAM and holistic health among adults in Jordan and identified factors associated with these beliefs. Methods: An online cross-sectional survey study was conducted between February and September 2025 using convenience sampling technique. Beliefs were measured with the Arabic version of the 11-item Holistic Complementary and Alternative Medicine Questionnaire (HCAMQ). Multivariable logistic regression compared participants with higher (≥median) and lower HCAMQ scores, and linear regression was conducted as a sensitivity analysis. Results: A total of 632 participants were involved in this study, of which 503 (79.6%) had heard of CAM, mainly through the media (50.7%). Among the 511 participants with complete HCAMQ data, the mean score was 52.74 ± 8.53, and 83.6% scored above the scale midpoint. Having heard of CAM was associated with higher scores (aOR = 2.16, 95% CI: 1.32–3.52), whereas diabetes mellitus (aOR = 0.34, 95% CI: 0.15–0.78) and current smoking (aOR = 0.56, 95% CI: 0.35–0.89) were associated with lower scores. Conclusions: Awareness of CAM among the general public in Jordan was high and beliefs were generally favourable in this predominantly young, female and educated sample. Public education on the evidence, benefits and risks of CAM is warranted.

## 1. Introduction

Complementary and alternative medicine (CAM), often known as “complementary health approaches,” is a collection of different healthcare practices and products that are not currently regarded as belonging to conventional medicine, but are frequently used with conventional medical care [1]. Types of CAM may include mind–body therapies, such as Quran recitations, and meditation, biologically based practices including vitamins, dietary supplements, and herbs, and body-based practices, such as massage therapy, and traditional Chinese medicine [2,3]. Some of these practices, such as Quran recitation, spiritual or faith healing and vitamin use, may not be considered as “alternative medicine” by lay people, which may influence how questions on CAM awareness are interpreted. The use of traditional, complementary and integrative medicine has become a global health phenomenon. According to the World Health Organization (WHO), traditional, complementary and integrative medicine is used in 170 countries, and findings from the WHO global survey indicate that 67% of responding countries reported use among 40–99% of their populations. This reflects the importance of understanding public beliefs and perceptions regarding CAM, due to the variability of the safety, quality, and effectiveness of different CAM practices [4]. These practices are often used to lessen side effects and discomfort associated with medical therapy, especially among patients with chronic diseases and cancer. Acupuncture, for instance, can be used by cancer patients to reduce the adverse effects of chemotherapy, such as nausea, vomiting, and pain [3]. Anxiety, tiredness, and pain can all be effectively reduced with massage therapy [5]. Some studies suggested that ginger may help reduce nausea and vomiting associated with chemotherapy and pregnancy [6]. It has also been found that yoga and meditation programs can help lower psychological stress [7,8]. Another form of CAM, art therapy, has been demonstrated to help women with cancer have better quality of life and experience less anxiety and depression [9].

Although CAM have many established advantages, using them without medical supervision can be dangerous and cause interactions with prescription drugs. Moreover, dietary supplements do not require approval from regulatory bodies for safety and effectiveness before they are marketed [10]. For example, one of the safety concerns associated with the use of herbal and dietary supplements is hepatotoxicity [11]. Previous studies have also shown that herbal remedies include bacteria and heavy metals, which can raise the risk of infections, metal toxicities, and even cancer [12]. Furthermore, lack of knowledge and awareness of the risks associated with these products, including interaction risks, further increases safety concerns regarding their use [13]. Another concern with CAM is the low proportion of patients who disclose their use to healthcare providers, as observed in a previous study, which reported that nearly 70% of cancer patients who used CAM said that they did not discuss their use with healthcare providers [14]. In Jordan, CAM use has also been documented across different populations and health conditions. A cross-sectional study involving 1057 Jordanians found generally positive attitudes toward medicinal plants, with 77.8% of participants believing in their efficacy [15]. A previous study in Jordan by Awwad et al. reported that 63.2% of females with polycystic ovary syndrome had used CAM, with herbal products being the most commonly used [16]. In addition, a recent study among students in Jordan found that more than two-thirds of participants were aware of CAM [17]. Previous studies largely focused on specific populations or specific type of CAM.

Despite evidence of CAM use in Jordan, limited research has examined CAM-related beliefs and holistic health perceptions among the general population. Therefore, this study aimed to evaluate awareness and beliefs regarding CAM and holistic health among the general public in Jordan and to identify factors associated with CAM-related beliefs.

## 2. Material and Methods

### 2.1. Study Design

This research was conducted in Jordan using an online cross-sectional survey study design between 13 February and 2 September 2025 using Qualtrics (XM version, Qualtrics, Provo, UT, USA). Data collection was extended over approximately seven months to reach the target sample size, because recruitment through social media relied on voluntary participation.

### 2.2. Study Population and Sampling Procedure

This study utilized convenience sampling to recruit eligible participants. Convenience sampling was selected because the study targeted members of the general Jordanian population through an online survey; however, no data on participants’ place of residence (governorate or urban/rural settings) were collected. Participation was limited to adults aged 18 and older who were currently living in Jordan. Social media platforms, specifically WhatsApp and Facebook, were used to solicit participants for the research. Individuals younger than 18 years and those who live outside Jordan were not eligible. The survey link was distributed by the research team and participation was restricted to one response per IP address. The total number of individuals who were invited to participate could not be estimated due to the utilization of convenience sampling; therefore, the response rate for this study could not be estimated.

### 2.3. Study Instrument

The Arabic version of a previously developed questionnaire tool by Hyland and Westoby was utilized in this study [18]. The questionnaire was reviewed by the research team to ensure that its wording was clear and appropriate for the Jordanian population. The questionnaire was translated using the forward–backward technique where it was translated from English into Arabic by one expert, and then another independent native Arabic speaker translated the questionnaire back to English. This was followed by comparing the back translated version with the original English one. Our study instrument comprises four sections. The first section focused on participants’ demographic characteristics utilizing multiple choice question (MCQ) format (age, gender, marital status, education level, monthly income, occupation, smoking, and comorbidities). The second section focused on source of information about CAM among participants utilizing MCQ and yes/no format questions. The third section examined awareness and perceived harmful effects of CAM utilizing MCQ and yes/no format questions. The last section examined participants’ beliefs about the scientific validity of CAM and holistic health using the 11-item Holistic Complementary and Alternative Medicine Questionnaire (HCAMQ) with a 7-point response scale ranging from strongly disagree (1) to strongly agree (7), including a neutral midpoint (4). The four negatively worded items (“The mind and body are separate entities”, “Complementary therapies pose a threat to public health”, “Complementary therapies rely on unscientific principles” and “Complementary therapies can be dangerous”) were reverse-scored, so that the total score ranged from 11 to 77, with higher scores indicating more positive beliefs about CAM and holistic health. The HCAMQ comprises a holistic health subscale (five items) and a CAM subscale (six items) [18]. The full questionnaire is provided as Supplementary File S1.

The HCAMQ was selected because it specifically assesses beliefs regarding the scientific validity of CAM and holistic health. The 11-item instrument has demonstrated acceptable psychometric properties and is therefore suitable for examining CAM-related beliefs. In this study, positive beliefs refer to greater acceptance of CAM and confidence in its potential value, whereas negative beliefs reflect greater skepticism or concerns regarding CAM. Holistic health refers to viewing health as involving physical, psychological, lifestyle, and other aspects of the individual.

### 2.4. Instrument Validation and Reliability

Face validity and clarity were assessed through a pilot study on 40 individuals who met the study eligibility criteria. The pilot participants were asked to complete the questionnaire and provide feedback regarding the clarity, wording, comprehensibility, and estimate completion time of the items. Based on their feedback, the questionnaire was reviewed for clarity and ease of completion before the main survey launched. Pilot participants were not included in the final study sample. The average time required to complete the questionnaire during the pilot study was approximately 5 min. Internal consistency was assessed with Cronbach’s alpha in the main study sample (*n* = 511). After reverse-scoring, Cronbach’s alpha was 0.68 for the 11-item scale, 0.68 for the holistic health subscale and 0.54 for the CAM subscale. The structural validity of the Arabic version was not assessed.

### 2.5. Sample Size

The minimum required sample size for this research was 383 individuals. This number was estimated using an expected proportion of 0.5, confidence interval of 95%, and a margin of error of 5%. A total of 632 participants completed the survey, of whom 511 had valid HCAMQ scores and were included in analyses involving the HCAMQ outcome. For the multivariable model, the smaller outcome group included 245 participants and 27 parameters were estimated, giving approximately nine events per parameter, which is slightly below the commonly recommended minimum of ten; the regression results should therefore be considered exploratory.

### 2.6. Data Analysis

Statistical methods were used to analyze the data. Descriptive statistics, including frequency and percentage, were calculated for categorical variables, and the mean and standard deviation (SD) for the HCAMQ score. Missing data arose because 20 participants stopped before the questions on CAM practice and health education, and a further 101 participants stopped before completing the final section (chronic conditions and HCAMQ). Descriptive statistics were therefore calculated using all available responses for each item), whereas the regression analyses were restricted to participants with complete data (complete-case analysis, *n* = 511). Multiple logistic regression was performed to identify factors associated with higher (more positive) beliefs toward CAM, measured by the HCAMQ scale, dichotomized at the sample median (53) as the dependent variable. Participants with a score of ≥53 were classified as having higher beliefs, and those with a score of <53 as having lower beliefs. The median score was selected as the cutoff because it provided a data-driven threshold that divided the population into two groups with relatively comparable distributions and allowed the factors associated with more positive versus less positive CAM beliefs to be examined using logistic regression. Because a median split defines the groups by construction, the proportion classified as having higher beliefs is not a prevalence estimate; the proportion of participants scoring above the theoretical scale midpoint (44, corresponding to a mean item score of 4, “neutral”) is therefore reported descriptively. Sociodemographic characteristics were included as independent variables. Because of sparse data, the 51–60 and ≥61 years age groups were combined and cancer (*n* = 6) was not included; the item “skin diseases”, which appeared twice in the questionnaire, was entered only once. The results of the multiple regression analyses are presented as adjusted odds ratios (aOR) with 95% confidence intervals (CI) and corresponding *p*-values; unadjusted odds ratios are provided in Supplementary Table S1. Model fit was assessed with the Hosmer–Lemeshow test and Nagelkerke R^2^. As sensitivity analyses, the model was refitted with the HCAMQ score as a continuous outcome (by applying multiple linear regression) and with the scale midpoint (>44) as the cut-off (Supplementary Table S2). Multicollinearity among the independent variables was assessed using variance inflation factor (VIF) and tolerance value. No problematic multicollinearity was identified, with VIF values ranging from 1.09 to 1.96 and tolerance values ranging from 0.51 to 0.92. The level of significance was defined as α = 0.05. No adjustment was made for multiple comparisons; the regression analyses were exploratory and should be interpreted accordingly. All calculations and analyses were carried out with IBM SPSS Statistics, version 31.0 (IBM Corp., Armonk, NY, USA).

## 3. Results

A total of 632 participants were included in the analysis. Regarding age distribution, most participants were aged 18–30 years (442; 69.9%). Most participants were female (516; 81.6%). The majority of the sample were single (379; 60.0%). Regarding education level, 466 participants (73.7%) had a bachelor’s degree. A total of 170 participants were active smokers (26.9%), and 61 (9.7%) had comorbidities. Additional details about demographic characteristics are provided in Table 1.

Regarding the complementary and alternative medicine, most participants (503; 79.6%) had heard of it. The primary source for information about CAM was media (255; 50.7%), followed by family, friends, or relatives (213; 42.3%). Traditional healers (58; 11.5%) were the least common source. Additional details about CAM are provided in Table 2.

The majority of the participants (446; 70.6%) are familiar with herbal medicine therapy as a form of complementary and alternative medicine, followed by Vitamins and other nutritional therapies (158; 25.0%) and massage therapy (130; 20.6%). Most participants (553; 90.4%) believed that education about the risks and benefits of CAM is important. Among the 553 participants who considered such education important, the most frequently reported harmful effects were abdominal pain (196; 35.4%) and diarrhea (185; 33.5%). Additional details about awareness and perceived harmful effects of CAM are provided in Table 3.

The majority of participants did not report having certain diseases, with 96.9% not having kidney diseases and 98.0% not having liver diseases. However, conditions such as diabetes mellitus (7.6%), hypertension (6.1%), and cardiovascular diseases (3.7%) were less common. The data indicated cancer was the least prevalent comorbidity, affecting only 1.2%. Further details about prevalence of comorbidities among participants are provided in Table 4.

When the three agreement categories (slightly agree, agree and strongly agree) were combined, most participants agreed that psychological well-being is a key factor in maintaining health (411; 80.4%), that harmony and balance within the body are important for health (397; 77.7%), and that a person’s thoughts can affect their physical health (395; 77.3%). Just over half of the study participants agreed that complementary therapies can be as effective as traditional medical treatments (287; 56.2%), add value to traditional medicine (282; 55.2%), and should be available in the national healthcare system (274; 53.6%). At the same time, 228 participants (44.6%) agreed that complementary therapies can be dangerous, 150 (29.4%) that they rely on unscientific principles, and 119 (23.3%) that they pose a threat to public health; neutral responses were common for these three items (29.9–35.8%). Additional details are provided in Table 5.

After reverse-scoring of the negatively worded items, the mean total HCAMQ score was 52.74 ± 8.53 (median 53, interquartile range 47–59; possible range 11–77), corresponding to a mean item score of 4.79 on the 7-point scale. Overall, 427 participants (83.6%) scored above the scale midpoint of 44. Using the median split, 266 participants (52.1%) had higher (≥53) and 245 participants (47.9%) had lower (<53) HCAMQ scores.

The multivariable logistic regression analysis findings are available in Table 6. Participants who had heard of CAM had higher odds of higher HCAMQ scores (aOR = 2.16, 95% CI: 1.32–3.52; *p* = 0.002), whereas participants with diabetes mellitus (aOR = 0.34, 95% CI: 0.15–0.78; *p* = 0.011) and current smokers (aOR = 0.56, 95% CI: 0.35–0.89; *p* = 0.014) had lower odds. No other variable was significantly associated with HCAMQ scores. The model showed adequate fit (Hosmer–Lemeshow χ^2^ = 11.23, *p* = 0.189; Nagelkerke R^2^ = 0.113). These associations were consistent when the HCAMQ score was analysed as a continuous outcome (linear regression, R^2^ = 0.10), in which a monthly income above 1500 JOD was associated with higher scores (B = 3.96, 95% CI: 0.40–7.51; *p* = 0.029); with the midpoint cut-off, diabetes and having heard of CAM remained significant (Supplementary Table S2). Unadjusted odds ratios are presented in Supplementary Table S1.

## 4. Discussion

The majority of participants in the current study were familiar with the concept of CAM (79.6%). The primary source for information about CAM was media (50.7%), followed by family, friends, or relatives (42.3%), while traditional healers were the least common source (11.5%). A study of university students in Jordan revealed that about 70% of participants knew about CAM and that they mostly used the internet to learn about it [17]. According to a different Jordanian study, cancer patients’ primary sources of information about CAM were friends and family, followed by the media [19]. Nearly 53% of respondents in a Saudi study knew something about CAM. While only 15.2% of individuals cited family, friends, or relatives as their primary source of CAM knowledge, about half cited the media as their primary source [20]. These results show that the internet is a major source of information regarding CAM, highlighting the importance of creating online awareness campaigns to increase public knowledge about CAM. However, depending on the internet, family, and friends for information about CAM might result in biased information, underscoring the significance of encouraging individuals to get such information from sources that have been scientifically validated.

About 70.6% of participants in the current survey classified herbal medicine as a sort of CAM, compared to 25% who identified vitamins and nutritional therapies and 20.6% who identified massage therapy as CAM modalities. Similarly, herbal medicine was the most recognized and utilized CAM modality among medical students in Palestine [21], although that sample comprised future healthcare professionals (medical students) rather than members of the general public. According to a systematic review, herbal medicine was the most popular form of CAM among patients and consumers in the UK [22]. Compared to vitamins and other CAM modalities, herbal medicine remained the most commonly recognized form of CAM across several other Middle Eastern countries [23,24,25]. Future public health programs should therefore concentrate on raising people’s knowledge of the entire range of CAM modalities, including the implementation of tailored educational campaigns about their proper utilization and safety issues. In fact, 90.4% of the participants in the current study agreed that it is crucial to educate people about the benefits and risks of CAM. This was highlighted in a systematic review that reported high levels of satisfaction towards educational interventions on CAM among consumers, patients, and caregivers [26]. The review also reported that these interventions were effective in improving CAM-related knowledge and decision-making skills.

Regarding the negative consequences of CAM, about one third of participants who answered this question expressed concerns about abdominal pain (35.4%) and diarrhea (33.5%). However, the response options for this question were limited to gastrointestinal symptoms and skin discoloration, so these findings do not capture the full range of potential CAM-related harms. These concerns can be explained by the evidence reported in the literature on the side effects of CAM. Digestive symptoms accounted for 44.3% of all reported adverse events from herbal medications in randomized controlled trials, followed by nervous system side effects (17.3%), according to a systematic review [27]. Only a small percentage of participants in a Thai study who reported taking dietary and herbal supplements experienced negative side effects. The most frequently reported adverse effects were gastrointestinal issues, most of which were mild [28,29]. Regarding the prevalence of comorbidities in this study, 90.3% of participants had no comorbid conditions. Nonetheless, 7.6%, 6.1%, and 3.7% of participants had diabetes mellitus, hypertension, and cardiovascular diseases, respectively. On the other hand, cancer was the least prevalent comorbidity, affecting only 1.2% of individuals. This low prevalence of comorbid conditions in this sample could be justified by the relatively young age of participants, given that more than two-thirds of them were 30 years of age or younger.

In this study, the mean HCAMQ score was 52.74 ± 8.53, and 83.6% of participants scored above the scale midpoint, indicating generally favourable beliefs about CAM and holistic health in this sample. In contrast, a largely neutral attitude towards CAM was reported among medical students in Saudi Arabia [30], although that sample is not directly comparable with the general public. Most participants in this survey thought that a person’s thoughts might have an impact on their physical health (77.3%) and that balance and harmony inside the body are crucial for good health (77.7%). Similarly, a study conducted in New Zealand on individuals with mental diseases revealed that the participants thought there was a link between mental and physical health [31]. These views are consistent with other studies that reported evidence of a link between mental and physical health [32,33,34]. More than half of the current study participants said complementary therapies were beneficial to traditional medicine (55.2%) and may be just as successful as traditional treatments (56.2%). In comparison, only 1% of respondents in a survey conducted in Saudi Arabia thought CAM had no therapeutic value, while the vast majority thought they were helpful [20]. Approximately two-thirds of participants in another study of oncology patients from Ireland thought that CAM was effective [35]. On the other hand, 44.6% of participants in the current survey felt that these therapies could be hazardous, and 23.3% said that CAM posed a threat to public health. In comparison, one-third of healthcare professionals in a previous study felt that CAM is dangerous and poses a threat to public health. Additionally, almost 72% of them acknowledged that laws and restrictions regarding the use of herbal medicines are necessary [36]. Furthermore, Polish doctors expressed more safety concerns, with 95% of them viewing CAM as harmful [37]; as these studies involved healthcare professionals, their findings are not directly comparable with those of the general public. These findings highlight the need for stronger public education on CAM safety, appropriate utilization, and potential interactions with conventional medicines. The positive beliefs observed may reflect the influence of cultural traditions, personal experiences, and information received through family friends, and media. However, these explanations are hypothetical, and future qualitative research is needed to better understand the factors shaping CAM beliefs among Jordanians.

Although counselling by healthcare providers was not assessed in this study, providers may have an important role in addressing CAM-related beliefs. Since only 18.1% of participants reported healthcare providers as a source of CAM information, pharmacists and physicians could routinely ask about CAM use and provide evidence-based advice on potential adverse effects, contraindication, and interactions with conventional medicines. Such counselling should be patient-centered and nonjudgmental to encourage disclosure and support informed decisions. The effectiveness of such counselling should be evaluated in future studies.

In the multivariable logistic regression analysis, participants who had heard of CAM had approximately twice the odds of higher HCAMQ scores, which is consistent with the expectation that familiarity with CAM is linked to more favourable beliefs; however, the direction of this association cannot be determined from a cross-sectional design. Participants with diabetes mellitus and current smokers had lower odds of higher scores. People with diabetes are often advised about blood glucose control and possible drug–herb interactions and may therefore be more cautious about CAM, but this explanation is speculative, particularly given the small number of participants with diabetes (*n* = 39). Monthly income was not associated with HCAMQ scores in the main analysis; previous studies of the relationship between income and CAM use have reported inconsistent results [38,39,40,41]. Because 27 parameters were estimated without adjustment for multiple comparisons, these associations should be regarded as exploratory and require confirmation in larger, representative samples.

This study has several limitations. First, the cross-sectional study design has limited ability to examine causality across the study variables. The sampling technique used (convenience sampling) and online recruitment may have introduced selection bias and reduced the generalizability of the study findings toward younger, more educated, and digitally connected individuals. In particular, the sample was predominantly female (81.6%) and highly educated (73.7% held a bachelor’s degree), 21.2% worked in the medical field, no data on place of residence were collected, and the results were not weighted to the Jordanian population. Besides, self-reported data is prone to recall bias and possible non-response and missing-data bias, the modest internal consistency of the Arabic HCAMQ in this sample (α = 0.68) and the lack of an assessment of its structural validity, and the inability to infer causality or actual CAM utilisation from an attitude scale. In addition, dichotomising the HCAMQ score at the median leads to a loss of information, although the main findings were consistent when the score was analysed as a continuous variable. Finally, the question on harmful effects offered a limited set of response options, and the item on skin diseases was inadvertently duplicated in the questionnaire. Therefore, the study findings should be interpreted carefully.

## 5. Conclusions

This study found that awareness of CAM was high among the general public in Jordan and that beliefs about CAM and holistic health were generally favorable. Having heard of CAM was associated with higher HCAMQ scores, whereas diabetes mellitus and current smoking were associated with lower scores; these exploratory associations require confirmation. Most participants considered education about the risks and benefits of CAM important, which supports public education that promotes evidence-based CAM use. Because the sample was predominantly young, female and educated, the findings may not be generalisable to the wider Jordanian population; future research should use representative sampling and assess actual CAM use.

## Figures and Tables

**Table 1:** Demographic characteristics of the participants.

Demographic Characteristics		N	%
Age (years)	18–23	222	35.1%
	24–30	220	34.8%
	31–40	112	17.7%
	41–50	51	8.1%
	51–60	26	4.1%
	Above 61	1	0.2%
Gender	Female	516	81.6%
	Male	116	18.4%
Marital status	Single	379	60.0%
	Married	237	37.5%
	Divorced	10	1.6%
	Widowed	6	0.9%
Education level	High school or less	104	16.5%
	Bachelor	466	73.7%
	Grad	62	9.8%
Monthly income (JOD)	Less than 500	275	43.5%
	500–1000	276	43.7%
	1000–1500	49	7.8%
	Above 1500	32	5.1%
Occupation	Retired	28	4.4%
	Not working	193	30.5%
	Medical field	134	21.2%
	Student	168	26.6%
	Other fields	109	17.2%
Smoking	No	462	73.1%
	Yes	170	26.9%
Comorbidities	No	571	90.3%
	Yes	61	9.7%

JOD: Jordanian dinar.

**Table 2:** Source of information about complementary and alternative medicine among participants (N = 632; sources of information *n* = 503).

Source of Information		N	%
Have you ever heard of CAM?	No	129	20.4%
	Yes	503	79.6%
If yes, what is the source of your information about complementary and alternative medicine? (*n* = 503) *	Family, friends, or relatives	213	42.3%
	Health care providers	91	18.1%
	Media (TV, Radio, Internet)	255	50.7%
	Patients who use complementary and alternative medicine.	100	19.9%
	Traditional healers.	58	11.5%
	Others	59	11.7%

* The total ≠ 100% due to multiple selection.

**Table 3:** Awareness and perceived harmful effects of complementary and alternative medicine (N = 632; items on combined practice and health education *n* = 612; harmful effects *n* = 553).

Awareness and Perceived Harmful Effects		N	%
What treatment options do you know from complementary and alternative medicine? *	Herbal medicine therapy	446	70.6%
	Spiritual/faith healing	81	12.8%
	Traditional bone-setting	54	8.5%
	Massage therapy	130	20.6%
	Vitamins and other nutritional therapies	158	25.0%
	Others	39	6.2%
Practicing with knowledge of both CAM and modern medicine is better than practicing with knowledge of modern medicine alone.	No	342	55.9%
	Yes	270	44.1%
Is health education about the risks and benefits of CAM important?	No	59	9.6%
	Yes	553	90.4%
If yes to the question, what are the harmful effects of complementary and alternative medicine? *	Diarrhea	185	33.5%
	Vomiting	136	24.6%
	Abdominal pain	196	35.4%
	Skin discoloration	73	13.2%
	Others	205	37.1%

* The total ≠ 100% due to multiple selection.

**Table 4:** Prevalence of comorbidities among participants (*n* = 511).

Comorbidities	No		Yes	
	N	%	N	%
**Diabetes Mellitus**	472	92.4%	39	7.6%
**Hypertension**	480	93.9%	31	6.1%
**Dyslipidemia**	465	91.0%	46	9.0%
**Cardiovascular diseases**	492	96.3%	19	3.7%
**Neurological diseases**	482	94.3%	29	5.7%
**Bone diseases**	475	93.0%	36	7.0%
**Skin Diseases**	464	90.8%	47	9.2%
**Kidney disease**	495	96.9%	16	3.1%
**Liver diseases**	501	98.0%	10	2.0%
**Respiratory diseases**	469	91.8%	42	8.2%
**Psychiatric disorders**	478	93.5%	33	6.5%
**Cancer**	505	98.8%	6	1.2%
**Others**	472	92.4%	39	7.6%

**Table 5:** Responses to HCAMQ scale items on complementary and alternative medicine beliefs (*n* = 511); values are *n* (%).

	Strongly Disagree	Disagree	Slightly Disagree	Neutral	Slightly Agree	Agree	Strongly Agree
The mind and body are separate entities.	189 (37.0)	79 (15.5)	37 (7.2)	100 (19.6)	35 (6.8)	56 (11.0)	15 (2.9)
A person’s thoughts can affect their physical health.	34 (6.7)	17 (3.3)	15 (2.9)	50 (9.8)	61 (11.9)	205 (40.1)	129 (25.2)
Spiritual beliefs can play a key role in maintaining health.	29 (5.7)	39 (7.6)	31 (6.1)	116 (22.7)	87 (17.0)	156 (30.5)	53 (10.4)
Harmony and balance within the body are important for health.	21 (4.1)	13 (2.5)	25 (4.9)	55 (10.8)	42 (8.2)	198 (38.7)	157 (30.7)
Psychological well-being is a key factor in maintaining health.	18 (3.5)	15 (2.9)	18 (3.5)	49 (9.6)	52 (10.2)	198 (38.7)	161 (31.5)
Complementary therapies pose a threat to public health.	48 (9.4)	82 (16.0)	79 (15.5)	183 (35.8)	60 (11.7)	43 (8.4)	16 (3.1)
Complementary therapies add value to traditional medicine.	22 (4.3)	28 (5.5)	37 (7.2)	142 (27.8)	91 (17.8)	152 (29.7)	39 (7.6)
Complementary therapies can be as effective as traditional medical treatments.	26 (5.1)	29 (5.7)	38 (7.4)	131 (25.6)	110 (21.5)	150 (29.4)	27 (5.3)
Complementary therapies should be available in the national healthcare system.	28 (5.5)	25 (4.9)	30 (5.9)	154 (30.1)	87 (17.0)	156 (30.5)	31 (6.1)
Complementary therapies rely on unscientific principles.	44 (8.6)	91 (17.8)	72 (14.1)	154 (30.1)	73 (14.3)	59 (11.5)	18 (3.5)
Complementary therapies can be dangerous.	31 (6.1)	41 (8.0)	58 (11.4)	153 (29.9)	117 (22.9)	86 (16.8)	25 (4.9)

**Table 6:** Multivariable logistic regression analysis of factors associated with higher HCAMQ scores (≥53) (complete-case analysis, *n* = 511).

Variables	Variable	aOR (95% CI)	*p* Value
Age (years)	18–23	Reference	
	24–30	0.85 (0.51–1.43)	0.548
	31–40	1.37 (0.72–2.63)	0.342
	41–50	1.33 (0.57–3.13)	0.515
	51 and above	1.16 (0.32–4.19)	0.824
Gender	Female	Reference	
	Male	1.30 (0.76–2.23)	0.345
Education level	High school or less	Reference	
	Bachelor	1.13 (0.62–2.05)	0.692
	Grad	1.06 (0.46–2.43)	0.899
Monthly income (JOD)	Less than 500	Reference	
	500–1000	1.14 (0.76–1.73)	0.521
	1000–1500	0.60 (0.29–1.23)	0.162
	Above 1500	2.40 (0.90–6.41)	0.081
Occupation	Not working	Reference	
	Retired	2.09 (0.67–6.56)	0.205
	Medical field	1.16 (0.66–2.05)	0.609
	Student	1.15 (0.66–2.01)	0.626
	Other fields	1.26 (0.68–2.35)	0.461
Smoking	No	Reference	
	Yes	0.56 (0.35–0.89)	0.014
Diabetes mellitus	No	Reference	
	Yes	0.34 (0.15–0.78)	0.011
Hypertension	No	Reference	
	Yes	1.79 (0.71–4.50)	0.216
Dyslipidemia	No	Reference	
	Yes	1.05 (0.49–2.28)	0.894
Cardiovascular diseases	No	Reference	
	Yes	0.47 (0.13–1.68)	0.242
Neurological diseases	No	Reference	
	Yes	1.27 (0.47–3.39)	0.638
Bone diseases	No	Reference	
	Yes	0.61 (0.25–1.48)	0.272
Skin diseases	No	Reference	
	Yes	0.92 (0.47–1.79)	0.798
Kidney diseases	No	Reference	
	Yes	0.56 (0.16–1.92)	0.352
Liver diseases	No	Reference	
	Yes	1.96 (0.38–9.98)	0.419
Respiratory diseases	No	Reference	
	Yes	0.84 (0.40–1.75)	0.643
Psychiatric disorders	No	Reference	
	Yes	0.94 (0.41–2.14)	0.873
Have you ever heard of CAM?	No	Reference	
	Yes	2.16 (1.32–3.52)	0.002

JOD: Jordanian dinar; aOR: Adjusted odds ratio; Hosmer–Lemeshow χ^2^ = 11.23, *p* = 0.189; Nagelkerke R^2^ = 0.113.

## Data Availability

The datasets used and/or analysed during the current study are available from the corresponding author on reasonable request.

## Supplementary Materials

The following supporting information can be downloaded at https://www.mdpi.com/article/10.3390/healthcare14213629/s1, File S1: Study questionnaire; Table S1: Unadjusted and adjusted odds ratios for higher HCAMQ scores (≥53) (complete-case analysis, *n* = 511); Supplementary Table S2: Sensitivity analyses: multiple linear regression of the continuous HCAMQ score and multivariable logistic regression using the scale midpoint (>44) as the cut-off (*n* = 511).
