# Supplementary material for: Public Awareness and Beliefs About Complementary and Alternative Medicine and Holistic Health in Jordan: A Cross-Sectional Survey
Source: Healthcare (Basel). 2026 Oct 26;14(21):3629. doi: 10.3390/healthcare14213629 (PMC13762484; doi:10.3390/healthcare14213629)
Supplement: Supplementary file 1 [file healthcare-14-03629-s001.zip › healthcare-4593599-supplementary.pdf]

Supplementary Material

Supplementary File S1. Study questionnaire.

نود دعوتكم للمشاركة في دراسة حول مدى استخدام الطب البديل في الأردن. تهدف هذه الدراسة إلى فهم جوانب وتفاصيل استخدامات الطب البديل في مجتمعنا، والعمل على تحسين الدعم والرعاية المقدمة للمرضى.

مشاركتكم في هذه الدراسة ستساعدنا على جمع بيانات هامة تساعد في تحسين جودة حياة المرضى. نؤكد لكم أن جميع المعلومات التي ستقدمونها ستعامل بسرية تامة وستستخدم فقط لأغراض البحث العلمي.

نحن بحاجة إلى صوتكم وآرائكم القيمة. نرجو منكم تخصيص بعض الوقت للمشاركة في هذا الاستبيان.

تعليمات: يرجى وضع إشارة (✓) في المربع المناسب. يمكن اختيار أكثر من إجابة فقط حيث يُذكر ذلك.

الجزء الأول: المعلومات الديموغرافية

1. العمر:

☐ 18 – 23 سنة ☐ 24 – 30 سنة ☐ 31 – 40 سنة

☐ 41 – 50 سنة ☐ 51 – 60 سنة ☐ 61 سنة فما فوق

2. الجنس:

☐ ذكر ☐ أنثى

3. الحالة الاجتماعية:

☐ أعزب / عزباء ☐ متزوج / متزوجة ☐ مطلق / مطلقة ☐ أرمل / أرملة

4. المستوى التعليمي:

☐ دراسة ثانوية أو أقل ☐ بكالوريوس ☐ دراسات عليا

5. الدخل الشهري للأسرة:

☐ أقل من 500 دينار ☐ 500 – 1000 دينار ☐ 1001 – 1500 دينار ☐ أكثر من 1500 دينار

6. الوضع الوظيفي:

☐ أعمل في المجال الطبي ☐ أعمل خارج المجال الطبي ☐ طالب جامعي

☐ لا أعمل ☐ متقاعد

7. هل تدخن في الوقت الحالي؟

☐ نعم ☐ لا

8. هل تعاني من أي أمراض مزمنة مثل أمراض القلب أو السكري أو الضغط أو ارتفاع مستوى الدهنيات أو غيرها؟

☐ نعم (أجب عن السؤال التالي) ☐ لا (انتقل إلى الجزء الثاني)

9. يرجى تحديد الأمراض المزمنة التي تعاني منها: *(لمن أجاب بـ "نعم" في السؤال 8)*

المرض	نعم	لا
السكري	☐	☐
الضغط	☐	☐
ارتفاع مستوى الدهنيات	☐	☐
أمراض القلب والأوعية الدموية	☐	☐
أمراض الأعصاب	☐	☐
أمراض العظام	☐	☐
أمراض جلدية	☐	☐
أمراض الكلى	☐	☐
أمراض الكبد	☐	☐
أمراض تنفسية	☐	☐
أمراض نفسية	☐	☐
سرطان	☐	☐
غير ذلك (يرجى التحديد): ____________________	☐	☐

الجزء الثاني: المعرفة والاتجاهات حول الطب التكميلي والبديل

10. هل سبق لك أن سمعت عن الطب البديل والتكميلي؟

☐ نعم ☐ لا

11. إذا كانت الإجابة بنعم، ما هو مصدر المعلومات عن الطب البديل والتكميلي؟ *(يمكن اختيار أكثر من إجابة)*

☐ العائلة والأصدقاء والأقارب ☐ مقدمو الرعاية الصحية

☐ وسائل الإعلام (الإنترنت، التلفزيون، الراديو، الكتب) ☐ المرضى الذين يستخدمون الطب البديل والتكميلي

☐ المعالجون التقليديون ☐ مصادر أخرى (حدد): ______________

12. ما هي خيارات العلاج التي تعرفها من الطب التكميلي والبديل؟ *(يمكن اختيار أكثر من إجابة)*

☐ العلاج بالأعشاب الطبية ☐ العلاج الروحي / الإيماني

☐ تقويم العظام التقليدي ☐ التدليك

☐ الفيتامينات والعلاجات الغذائية الأخرى ☐ علاجات أخرى (حدد): ______________

13. هل تعتقد أن الممارسة بمعرفة الطب التكميلي والبديل والطب الحديث معاً أفضل من الممارسة بمعرفة الطب الحديث فقط؟

☐ نعم ☐ لا

14. هل تعتقد أن التثقيف الصحي حول مخاطر وفوائد الطب البديل والتكميلي مهم؟

☐ نعم ☐ لا

15. ما هي الآثار الضارة للطب التكميلي والبديل التي تعرفها؟ *(يمكن اختيار أكثر من إجابة)*

☐ الإسهال ☐ القيء

☐ آلام البطن ☐ تغير لون الجلد

☐ أخرى (حدد): ____________

الجزء الثالث: المعتقدات الخاصة بالطب التكميلي والبديل

يرجى بيان مدى موافقتك على كل عبارة من العبارات التالية بوضع إشارة (✓) في المربع المناسب:

العبارة	أوافق بشدة	أوافق	أوافق قليلاً	محايد	أختلف قليلاً	أختلف	أختلف بشدة
1. العقل والجسم كيانان منفصلان.	☐	☐	☐	☐	☐	☐	☐
2. يمكن أن تؤثر أفكار الشخص على صحة جسده.	☐	☐	☐	☐	☐	☐	☐
3. يمكن أن تلعب المعتقدات الروحية دوراً رئيسياً في الحفاظ على الصحة.	☐	☐	☐	☐	☐	☐	☐
4. الانسجام والتوازن داخل الجسم مهمان للصحة.	☐	☐	☐	☐	☐	☐	☐
5. الرفاه النفسي عامل رئيسي في الحفاظ على الصحة.	☐	☐	☐	☐	☐	☐	☐
6. العلاجات التكميلية تشكل تهديداً للصحة العامة.	☐	☐	☐	☐	☐	☐	☐
7. العلاجات التكميلية إضافة قيمة إلى الطب التقليدي.	☐	☐	☐	☐	☐	☐	☐
8. يمكن أن تكون العلاجات التكميلية فعالة مثل العلاجات الطبية التقليدية.	☐	☐	☐	☐	☐	☐	☐
9. يجب أن تكون العلاجات التكميلية متاحة في النظام الصحي الوطني.	☐	☐	☐	☐	☐	☐	☐
10. العلاجات التكميلية تعتمد على مبادئ غير علمية.	☐	☐	☐	☐	☐	☐	☐
11. العلاجات التكميلية يمكن أن تكون خطيرة.	☐	☐	☐	☐	☐	☐	☐

Supplementary Table S1. Unadjusted and adjusted odds ratios for higher HCAMQ scores (≥ 53) (complete-case analysis, n = 511).

Variable	Category	cOR (95% CI)	p-value	aOR (95% CI)	p-value
Age (reference group: 18–23)	24–30	0.78 (0.52–1.18)	0.247	0.85 (0.51–1.43)	0.548
	31–40	1.16 (0.69–1.95)	0.568	1.37 (0.72–2.63)	0.342
	41–50	1.05 (0.54–2.04)	0.878	1.33 (0.56–3.12)	0.515
	51 and above	1.17 (0.47–2.91)	0.736	1.16 (0.32–4.19)	0.824
Gender (reference group: female)	Male	1.11 (0.71–1.75)	0.636	1.30 (0.75–2.23)	0.345
Education (reference group: high school or less)	Bachelor	1.34 (0.82–2.19)	0.245	1.13 (0.62–2.05)	0.692
	Postgraduate	1.22 (0.59–2.53)	0.589	1.06 (0.46–2.43)	0.899
Monthly income, JOD (reference group: < 500)	500–1000	1.23 (0.84–1.78)	0.284	1.14 (0.76–1.73)	0.521
	1000–1500	0.69 (0.35–1.36)	0.284	0.60 (0.29–1.23)	0.162
	Above 1500	2.29 (0.96–5.50)	0.063	2.40 (0.90–6.41)	0.081
Occupation reference group: not working)	Retired	2.02 (0.80–5.08)	0.137	2.09 (0.67–6.56)	0.205
	Medical field	1.27 (0.77–2.07)	0.349	1.16 (0.66–2.05)	0.609
	Student	1.35 (0.85–2.14)	0.199	1.15 (0.66–2.01)	0.626
	Other fields	1.45 (0.86–2.45)	0.167	1.26 (0.68–2.35)	0.461
Smoking (reference group: no)	Yes	0.67 (0.45–1.00)	0.050	0.56 (0.35–0.89)	0.014
Diabetes mellitus (reference group: no)	Yes	0.38 (0.19–0.77)	0.007	0.34 (0.15–0.78)	0.011
Hypertension (reference group: no)	Yes	1.13 (0.54–2.34)	0.749	1.79 (0.71–4.50)	0.216
Dyslipidemia (reference group: no)	Yes	0.83 (0.45–1.52)	0.548	1.05 (0.49–2.28)	0.894
Cardiovascular (reference group: no)diseases	Yes	0.41 (0.15–1.10)	0.077	0.47 (0.13–1.68)	0.242
Neurological (reference group: no)diseases	Yes	0.74 (0.35–1.56)	0.424	1.27 (0.47–3.39)	0.638
Bone diseases (reference group: no)	Yes	0.56 (0.28–1.13)	0.105	0.60 (0.25–1.48)	0.272
Skin diseases (reference group:	Yes	0.87 (0.48–1.59)	0.654	0.92 (0.47–1.79)	0.798

no)					
Kidney diseases (reference group: no)	Yes	0.54 (0.19–1.52)	0.243	0.56 (0.16–1.92)	0.352
Liver diseases (reference group: no)	Yes	0.92 (0.26–3.22)	0.896	1.96 (0.38–9.98)	0.419
Respiratory (reference group: no)diseases	Yes	0.67 (0.35–1.26)	0.215	0.84 (0.40–1.75)	0.643
Psychiatric disorders (reference group: no)	Yes	0.66 (0.32–1.35)	0.255	0.93 (0.41–2.14)	0.873
Heard of CAM (reference group: no)	Yes	2.12 (1.34–3.34)	0.001	2.16 (1.32–3.52)	0.002

cOR: crude (unadjusted) odds ratio; aOR: adjusted odds ratio. Hosmer–Lemeshow $\chi^2 = 11.23$, $p = 0.189$; Nagelkerke $R^2 = 0.113$. Cancer (n = 6) was excluded; JOD: Jordanian dinar.

Supplementary Table S2. Sensitivity analyses: multiple linear regression of the continuous HCAMQ score and multivariable logistic regression using the scale midpoint (> 44) as the cut-off (n = 511).

Variable	Category	Linear regression B (95% CI)	p-value	Midpoint cut-off aOR (95% CI)	p-value
Age (reference group: 18–23)	24–30	1.25 (-0.83 to 3.32)	0.238	1.75 (0.88–3.47)	0.111
	31–40	1.98 (-0.60 to 4.56)	0.131	1.36 (0.58–3.17)	0.477
	41–50	2.43 (-0.94 to 5.80)	0.158	1.71 (0.57–5.15)	0.343
	51 and above	4.02 (-0.83 to 8.88)	0.104	2.61 (0.56–12.07)	0.221
Gender (reference group: female)	Male	0.60 (-1.52 to 2.73)	0.577	0.97 (0.48–1.99)	0.943
Education (reference group: high school or less)	Bachelor	1.14 (-1.22 to 3.51)	0.341	1.59 (0.79–3.21)	0.192
	Postgraduate	0.31 (-2.97 to 3.59)	0.851	1.65 (0.56–4.81)	0.362
Monthly income, JOD (reference group: < 500)	500–1000	0.83 (-0.82 to 2.49)	0.323	0.98 (0.56–1.72)	0.954
	1000–1500	-0.27 (-3.11 to 2.58)	0.853	0.92 (0.36–2.40)	0.871
	Above 1500	3.95 (0.40 to 7.51)	0.029	2.87 (0.55–14.89)	0.209
Occupation (reference group: not working)	Retired	0.57 (-3.84 to 4.97)	0.801	0.93 (0.26–3.33)	0.916
	Medical field	0.58 (-1.70 to 2.85)	0.618	1.85 (0.84–4.07)	0.126
	Student	1.89 (-0.34 to 4.13)	0.096	2.00 (0.96–4.17)	0.062
	Other fields	1.47 (-1.02 to 3.95)	0.247	2.16 (0.89–5.22)	0.089
Smoking (reference group: no)	Yes	-1.91 (-3.76 to -0.05)	0.044	0.63 (0.34–1.16)	0.137
Diabetes mellitus (reference group: no)	Yes	-5.08 (-8.17 to -2.00)	0.001	0.32 (0.13–0.75)	0.009
Hypertension (reference group: no)	Yes	-0.41 (-3.88 to 3.05)	0.815	0.86 (0.29–2.58)	0.792
Dyslipidemia (reference group: no)	Yes	-1.29 (-4.30 to 1.73)	0.401	0.88 (0.33–2.37)	0.806
Cardiovascular diseases (reference group: no)	Yes	0.24 (-4.59 to 5.06)	0.923	0.57 (0.13–2.41)	0.444
Neurological diseases (reference	Yes	1.94 (-1.86 to 5.74)	0.316	3.72 (0.76–18.23)	0.105

group: no)					
Bone diseases (reference group: no)	Yes	-2.17 (-5.63 to 1.29)	0.219	0.66 (0.23–1.91)	0.444
Skin diseases (reference group: no)	Yes	-0.15 (-2.85 to 2.54)	0.912	0.70 (0.29–1.66)	0.413
Kidney diseases (reference group: no)	Yes	-3.36 (-8.16 to 1.44)	0.170	0.99 (0.20–4.82)	0.985
Liver diseases (reference group: no)	Yes	-0.43 (-6.63 to 5.77)	0.892	1.42 (0.19–10.78)	0.734
Respiratory diseases (reference group: no)	Yes	-0.48 (-3.36 to 2.40)	0.742	1.09 (0.40–2.95)	0.870
Psychiatric disorders (reference group: no)	Yes	2.53 (-0.71 to 5.78)	0.126	0.97 (0.34–2.79)	0.952
Heard of CAM (reference group: no)	Yes	3.15 (1.23 to 5.07)	0.001	2.04 (1.14–3.65)	0.017

Linear regression: $R^2 = 0.10$. Midpoint model: 427 participants scored above and 84 at or below the midpoint (44). B: unstandardised regression coefficient; aOR: adjusted odds ratio; JOD: Jordanian dinar.
